# Empathy Variation in General Practice: A Survey among General Practitioners in Denmark

**DOI:** 10.3390/ijerph15030433

**Published:** 2018-03-02

**Authors:** Justin A. Charles, Peder Ahnfeldt-Mollerup, Jens Søndergaard, Troels Kristensen

**Affiliations:** 1 Center for Medical Humanities, Compassionate Care, and Bioethics, Stony Brook University, Stony Brook, New York, NY 11794, USA; Justin.Charles@stonybrookmedicine.edu; 2 Research Unit of General Practice, Department of Public Health, University of Southern Denmark, J.B. Winsløws Vej 9, DK-5000 Odense C, Denmark; pahnfeldt-mollerup@health.sdu.dk (P.A.-M.); JSoendergaard@health.sdu.dk (J.S.); 3 COHERE, Department of Public Health & Research Unit of General Practice, University of Southern Denmark, 5000 Odense C, Denmark

**Keywords:** physician empathy, organization of care, medical education, general practice, variation, job satisfaction, Jefferson scale of empathy (JSE), GP characteristics, Denmark, primary care

## Abstract

*Background*: Previous studies have demonstrated that high levels of physician empathy may be correlated with improved patient health outcomes and high physician job satisfaction. Knowledge about variation in empathy and related general practitioner (GP) characteristics may allow for a more informed approach to improve empathy among GPs. *Objective*: Our objective is to measure and analyze variation in physician empathy and its association with GP demographic, professional, and job satisfaction characteristics. *Methods*: 464 Danish GPs responded to a survey containing the Danish version of the Jefferson Scale of Empathy for Health Professionals (JSE-HP) and questions related to their demographic, professional and job satisfaction characteristics. Descriptive statistics and a quantile plot of the ordered empathy scores were used to describe empathy variation. In addition, random-effect logistic regression analysis was performed to explore the association between empathy levels and the included GP characteristics. *Results*: Empathy scores were negatively skewed with a mean score of 117.9 and a standard deviation of 10.1 within a range from 99 (p5) to 135 (p95). GPs aged 45–54 years and GPs who are not employed outside of their practice were less likely to have high empathy scores (≥120). Neither gender, nor length of time since specialization, length of time in current practice, practice type, practice location, or job satisfaction was associated with odds of having high physician empathy. However, odds of having a high empathy score were higher for GPs who stated that the physician-patient relationship and interaction with colleagues has a high contribution to job satisfaction compared to the reference groups (low and medium contribution of these factors). This was also the trend for GPs who stated a high contribution to job satisfaction from intellectual stimulation. In contrast, high contribution of economic profit and prestige did not contribute to increased odds of having a high empathy score. *Conclusions*: Albeit generally high, we observed substantial variation in physician empathy levels among this population of Danish GPs. This variation is positively associated with values of interpersonal relationships and interaction with colleagues, and negatively associated with middle age (45–54 years) and lack of outside employment. There is room to increase GP physician empathy via educational and organizational interventions, and consequently, to improve healthcare quality and outcomes.

## 1. Introduction

Empathy in the clinical setting has been described as a multidimensional concept that encompasses cognitive, affective, moral, and behavioral components [1]. Some experts argue that empathy is primarily determined by heritability and early life experiences and therefore cannot be changed [2]. Others believe that a person’s environment and relationships throughout life can alter a person’s empathic capabilities under the right circumstances [3,4].

Empathy plays an important role in the relationship between the patient and general practitioner (GP), as it facilitates the trust and understanding that allows for effective communication of medical information and reduces emotional burden in both parties [5,6]. Previous studies have demonstrated a relationship between high levels of physician empathy and improved patient health outcomes, increased patient and physician satisfaction, and decreased physician burnout [7,8,9]. Nevertheless, patient reports indicate that there may be a gap between the desired and actual levels of empathy in this relationship, due in part to individual variation in the empathic capabilities of GPs [10,11].

The extent of variation and factors influencing such variation in the GP population are not well understood, but may include an individual’s demographic characteristics, such as age and gender, professional characteristics, such as practice type and clinical experience, and job satisfaction [12,13]. Analyzing the extent of this variation and its association with GP characteristics may inform more targeted educational and organizational interventions to improve empathy in primary care.

Such interventions are needed now more than ever due to the progressive decline in empathy that has been observed among younger people in recent years [14]. Proposed reasons for the decline include an increased pervasiveness in technology, which has reduced face-to-face communication, and an increase in narcissistic personality traits [14,15]. This, coupled with the documented decrease in student empathy that often occurs throughout medical school, has made improving empathy become an important issue in the field of medical education [16]. To address this issue, medical schools, such as University of Southern Denmark, have started to introduce narrative courses in attempts to improve interpersonal skills, including empathy, among future physicians. Courses in communication skills, literature, and art have been incorporated to improve empathy in both practicing physicians and medical students with promising initial results [17,18]. To the best of our knowledge, there is no systematic, formal empathy training among practicing physicians.

In this study, we aim to measure and analyze variation in physician empathy among Danish GPs, and to explore associations between selected GP characteristics and physician empathy. We focus on the cognitive and behavioral components of physician empathy. This perspective allows physician empathy to be viewed as a professional communication skill that can be learned, rather than as an innate, emotional experience [19]. Therefore, this paper takes the angle that empathy involves “the ability to understand a patient’s inner experiences and perspective”, as well as, “a capability to communicate this understanding” [20]. This focus differs from the affective component of empathy, which refers to the ability to share in a patient’s feelings [21].

First, we hypothesize that Danish GPs will have similarly high empathy levels to other studied GP populations, and higher empathy levels than more “technology-oriented” specialists [20,22]. Next, we postulate that part of the variation in physician empathy can be explained by demographic, professional (e.g., practice type), and job satisfaction characteristics. Moreover, we expect that a set of factors contributing to job satisfaction will have a stronger association with empathy than demographic or professional characteristics. For instance, we expect a positive association between empathy and a strong contribution of the physician patient relationship to GP job satisfaction, as factors that contribute to job satisfaction are expected to reflect an individual’s personality [23,24].

## 2. Methods

### 2.1. Survey

A web-based survey was sent to GPs currently practicing in Denmark. The survey included the Jefferson Scale of Empathy for Health Professionals (JSE-HP) to measure physician empathy. The JSE-HP is a self-report psychometric tool that measures a physician’s empathic behavioral orientation. It measures mainly cognitive and behavioral empathy through components of “perspective taking”, “compassionate care”, and “standing in the patient’s shoes”. It features 20 statements using a 7-point Likert scale (1 = strongly disagree, 7 = strongly agree), such as “I try to understand what is going on in my patients’ minds by paying attention to their nonverbal cues and body language”. Scores range from 20 to 140, where a higher score indicates a more empathic behavioral orientation. Originally created in English, it has been translated into 55 languages, including Danish. Evidence of its convergent, discriminant, concurrent, and predictive validity, as well as internal consistency, test-retest reliability, and low social desirability bias is well established among health professionals in the United States and to varying degrees in international settings, including Denmark [20,25,26,27,28]. Minor changes were made to the Danish version of the scale to better reflect the meaning of the English version. These were tested in a pilot study to a sample of Danish GPs before being implemented in the final version of the survey.

In addition, an addendum to the JSE-HP was created to capture information about GP characteristics and determine their potential association with physician empathy. The following characteristics were included: (a) demographics (gender, age), (b) professional experience (time since specialization, time spent practicing in current clinic, practice type, practice location, employment outside of GP practice) and (c) strength of selected factors contributing to job satisfaction: (c1) physician-patient relationship, (c2) intellectual stimulation, (c3) interaction with colleagues, (c4) economic profit, and (c5) prestige, as well as job satisfaction in general.

Gender was included as female physicians often have higher self-reported empathy scores than do males [29,30,31]. We expected this to remain true in our study population. Age was included as cognitive empathy may decrease with older age [32]. We predict a similar trend will exist in this study. In addition, empathy levels have been shown to increase with clinical experience (independent of age) and with longitudinal patient relationships [33,34]. Therefore, time since GP specialization and time spent practicing in current practice were included as measures of clinical experience and continuity of care respectively. 

Practice type was functionally split in our study between “partnership practices”, which share patients among GPs, and “non-partnership practices” which do not. The increased autonomy and continuity of care in the latter may affect empathy through its relation to better maintenance of long-term relationships [35]. Therefore, we expect GPs in non-partnership practices to have higher empathy than those in partnership practices. GPs from rural areas have been reported to have lower empathy than their urban counterparts [36]. We expect the same will hold true among Danish GPs.

Physicians who engage in activities with a prosocial and/or altruistic element (e.g., clinical supervisors or health care administrators) outside of their clinic have been shown to have higher empathy levels than their peers [37,38]. Furthermore, it has been claimed that social work practitioners who are more empathic are also more effective and can better balance their roles [39]. Therefore, GPs in our population with employment outside of their clinic are expected to have higher empathy levels.

Job satisfaction was measured by a question that asked how satisfied the GP was with their job, with five response options ranging from very unsatisfied to very satisfied. Previous studies demonstrated a bidirectional relationship between job satisfaction and empathy [12,40]. Finally, GPs were asked to rank how much the following factors contributed to their job satisfaction on a 7-point Likert scale: physician-patient relationship, prestige, intellectual stimulation, interaction with colleagues, and economic profit. These factors were chosen because they are commonly implicated as important to a physician’s job satisfaction [41,42]. Interactions with patients and with empathetic colleagues has been shown to promote empathy among medical professionals [43]. Therefore, one would expect a correlation between empathy levels and a high contribution of the physician-patient relationship and interaction with colleagues to GP satisfaction.

### 2.2. Sample Selection

The survey contained 20 covariates. Based on this number, the preferred sample size was about 400 GPs. We aimed to forward the survey to around 1200 GPs with the expectation of a 30–50% response rate [44]. The sample was selected from a list of 2926 email addresses of GPs from the General Practitioners Organization (PLO, Copenhagen, Denmark) from 2013. GPs who have stopped working were excluded. Empirical evidence indicates that GPs may have different characteristics across practice types and locations [8,45]. To address this, a “stratified proportion allocation” was applied to a random sample of GPs from different subpopulations. Six strata were created based upon combinations of practice type (partnership, non-partnership) and practice location (urban, rural, mixed urban-rural), and are shown below. Categorization of the municipality type and practice type was based on a 2011 report and PLO registry data respectively [46]. 71.05% of the 2926 GPs were from partnership practices and 28.95% were from non-partnership practices. The distribution of age intervals was as follows: 10.8% (35–44 years); 33.3% (45–54 years); 42.7% (55–64 years) and 13.3% (65+ years). In addition, 48.4% were females.

To create a random stratum distribution, we first determined the proportion of the 2926 GPs that fit each of the six strata. Then, we multiplied this proportion to our desired number of survey recipients (*n* = 1200). Random numbers in the interval [0,1] were assigned to the GPs in each stratum. These were then ordered, and GPs with a number below the calculated relative proportion from the stratum were included in the sample. This produced the random strata distribution of 1195 GPs that is displayed in Table 1 below.

In December 2016, our survey was emailed to these 1195 Danish GPs using SurveyXact software, accompanied by a cover letter describing the study. Participants were offered the equivalent of $20 for their time as per protocol of the Danish Multipractice Committee who approved our study. Two reminder emails were sent to non-respondents 2 and 4 weeks after the initial email. This study received ethical approval from the Institutional Review Board at Stony Brook University.

### 2.3. Statistical Analysis

Descriptive statistics and a quantile plot of the ordered empathy scores were used to describe empathy variation. To exploit the benefits of using a binary dependent variable, we applied a random-effect logistic regression model to examine the contribution of the included GP characteristics [47]. A split of the empathy score (*ES*) was performed at a score of 120 to create the dichotomized dependent variable representing “high-scorers” (score of ≥120) and “low-scorers” (score <119). The model takes the following form:

(1) $$ES_{ij}^{*}=\beta_{00}+\beta^{\prime }x_{ij}+u_{j}+\varepsilon_{ij}$$

where the dependent dummy variable *ES_ij_* was defined by:

(2) $$ES_{ij}=\{\begin{array}{c} \;1\;\;\;if\;ES\;\geq 120\\ 0\;\;\;\mathrm{otherwise} \end{array}$$

The parameter $x_{ij}$ in Equation (1) is a row vector of explanatory variables containing characteristics of respondent i = 1…*n* in clinic *j*. The term *u_j_* is the random effect of being in group *j* where *u_j_* ~ N (0, σ_u_^2^). *β* represents within group change. This model allows the probability to vary from clinic to clinic and ε_ij_ is the residual at respondent level.

Covariates with responses of binary nature, such as gender, practice type, and employment outside of clinic, were coded with dummy variables. We also split the following variables into groups: age; years since GP specialization; years in present practice; job satisfaction characteristics. A Wald test was used to test the overall significance of the model. The intra-class correlation coefficient was used to estimate the proportion of overall residual variability linked to the clinic level. The variance inflation factor (VIF) was used to measure the extent of multi-collinearity among covariates, signified by a VIF > 10. Since there is potential for correlated observations among providers within the same practice, random-effect was used. All analyses were performed using Stata Version 14 (Stat/IC, College Station, TX, USA).

To analyze over- and under-representativeness of the respondents with respect to the stratification criteria, the ratio between the proportions of respondents who belong to each stratum in the sample and the population was calculated. A value over 1.00 reflects overrepresentation and vice versa. The representativeness of gender and age groups was also assessed via these ratios. Self-reported physician age and gender were compared to registry data to determine the validity of the survey responses and correct discrepancies. Cronbach’s alpha was calculated for the JSE-HP to report its internal consistency reliability [48].

## 3. Results

Of the 1195 GPs who were sent a copy of the survey, a total of 464 GPs from 406 practices completed the entire questionnaire (response rate of 38.8%). There was a minimum of one and maximum of three GPs from any one practice, with an average of 1.1 GPs per practice.

The descriptive statistics for the 464 GP respondents’ demographic and clinic characteristics are shown in Table 2. The average GP respondent is 54.9 years old and 53.4% are males. Most of the respondents work in partnership practice (72%) and 49.3% work in urban locations. The average GP has been specialized for 19 years and has been in his or her current practice for 17 years.

Table 2 also displays descriptive statistics of the GPs’ scores from the JSE-HP. The scores varied from 80 to 140. Mean and median scores were 117.8 and 118 respectively. The extent of variability of the scores is shown via standard deviation (SD), coefficient of variation (CV) and the 5th and 95th percentiles (p5 and p95). The distribution was negatively skewed and mesokurtic (skewness = −0.37, kurtosis = 2.83) No GPs scored in the range from 20 to 80. Cronbach’s alpha was 0.85, indicating internal consistency. Over half (54.5%) of GPs have additional employment outside of their main clinic, such as research, teaching, and political activity.

71.5% of the respondents are from partnership practices (13.8% points rural, 25.4% points mixed, 32.3% points urban municipalities) and 28.4% are from non-partnership practices (3.4% points rural, 8% points mixed, 17% points urban). The representativeness-ratio for each of the strata are as follows: (a) 0.575 (non-partnership and rural); (b) 1.02 (non-partnership and mixed); (c) 1.12 (non-partnership and urban); (d) 0.88 (partnership and rural); (e) 0.77 (partnership and mixed); (f) 1.44 (partnership and urban). This shows an overrepresentation of respondents in partnership practices in urban locations and an underrepresentation of respondents in partnership practices in heterogeneous municipality locations. The distribution of age intervals are as follows: 11.4% (35–44 years); 36.0% (45–54 years); 41.8% (55–64 years); 10.78% (65+ years). The representativeness ratios for age ratios are as follows: 1.06 (35–44 years); 1.09 (45–54 years); 0.98 (55-64 years); 1.23 (65+ years). Therefore, there was no significant difference between the ages of those who responded to the survey and those who did not.

**Figure 1:**
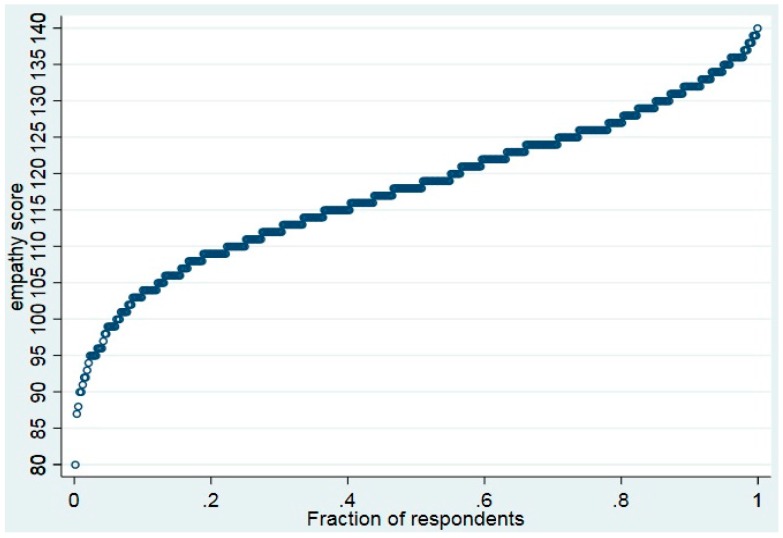
Ordered values of the empathy score versus fractions of respondents.

Figure 1 shows the variation in empathy score across the ordered values of GP respondents. For instance, 20% of respondents scored ≤108 and 20% scored ≥127. A histogram of empathy scores also demonstrates a negative skew of this data (not included). Thus, the results demonstrate variation in empathy scores among different subsets of respondents.

The results regarding job satisfaction are shown in Table 3. Most respondents (79.7%) are at least somewhat satisfied with their jobs as GP, while only 9.9% are somewhat or very unsatisfied. GPs’ responses regarding contribution of medical practice factors to job satisfaction show that the physician-patient relationship contributed the most to their job satisfaction (6.71/7). Intellectual stimulation (5.65/7) and interaction with colleagues (5.41/7) were also rated as important to GPs’ job satisfaction. In contrast, economic profit (4.92/7) and prestige contributed the least (3.71/7). Table 4 summarizes results of the logistic regression in terms of odds ratios (OR). Overall, the model shows that a high empathy score is associated with a part of the GP characteristics among respondents in the sample.

Male GPs did not have higher odds of having a high empathy score than did female GPs. GPs in the age range of 45–54 years had a 56% decrease in odds of having a high empathy score compared to the youngest age group, indicated by an OR of 0.44 (*p* = 0.036). A greater number of years since GP specialization or in present practice did not influence the odds of having higher empathy. This lack of association also existed for practice locations and practice types, which do not impact the odds of having a high empathy score. In contrast, GPs who are not employed outside of clinic had a 41% decrease in odds of having high empathy compared to the reference group with employment outside of clinic (OR = 0.59; *p* = 0.016). Physicians who believe that the physician-patient relationship (OR = 4.30; *p* < 0.0001) and interaction with colleagues (OR = 1.90; *p* = 0.006) are of high importance to their job satisfaction had significantly higher odds of having a high empathy score. Those who view intellectual stimulation as having high importance to job satisfaction had an increased OR that was slightly below significant (1.53; *p* = 0.053). The estimated intra-class correlation coefficient revealed that the overall residual variability was not significant association with the clinic level. The variance inflation factor (VIF) did not reveal multi-collinearity.

## 4. Discussion

Mostly consistent with our predictions, empathy scores in our study population (mean: 117.8) were similar to those found in previous studies of Danish GPs (mean: 117.2) [28] and American primary care doctors (mean: 116.6) [49], and slightly, but not significantly, higher than those found in French GPs (mean: 111.8) [37], American diagnostic radiologists (mean: 110.7) [31], American urologists (mean: 113.8) [31] and Korean technology-oriented specialists (mean: 106.9) [30]. A possible explanation for this phenomenon is that medical students with higher levels of empathy choose specialties that involve a greater deal of interpersonal interaction [50].

There was variation among physician empathy in our study population, indicated by a wide range (80–140) of scores that were negatively skewed, with a near even split between high and low-scorers. This signifies that, as hypothesized, there is a subset of Danish GPs in which empathy levels can ideally be increased. Specific interventions, such as mindfulness interventions, communication skills training and standardized patient encounters can increase empathy in medical students and physicians [49,51,52]. Several programs were assessed using reliable and valid outcome measures, randomized controlled trials, and large sample sizes [17]. Improvements in empathy may even be sustained after the termination of an intervention [49]. Initial research demonstrates that efficacy of empathy training programs can be improved by providing compensation for participation and focusing on cognitive and behavioral components of empathy [53]. These findings can aid in the development of formal, organized empathy training to be used on an ongoing basis in both undergraduate and continuing medical education.

One of the goals of this study was to determine what factors among a set of observable GP characteristics are associated with GP empathy variation, which could inform future attempts to develop empathy interventions targeted for GPs. Our results indicated a negative association between physician age of 45–54 years and being a high empathy scorer, yet no association between empathy and length of time since specialization, as a measure of clinical experience. Based on the results of prior demographic studies, we expected cognitive empathy to decrease with age [32]. However, others have found that this relationship did not hold when the personal relevance of the task was controlled for [54]. Our findings may instead be related to increasing levels of burnout in the medical field [55]. Another explanation is that this age group may have increased stressors outside of work, which decrease their capacity for empathy [11]. The relationship between age and empathy is likely multidirectional and context-dependent [56]. Future studies involving a longitudinal analyses of empathy levels in physicians over their careers may help better explain these results.

There was no association between gender and physician empathy in our study, which was consistent with several other studies using the JSE-HP [29,57]. However, several studies suggest that gender differences in empathy in favor of females are relatively consistent [58,59]. In addition, other literature describes that female physicians have higher empathy scores than do male physicians [29,30,31]. Perhaps the absence of a relationship in our study is related to our use of the JSE-HP, as gender differences are less commonly found when using this scale than when using other measures of empathy. Future studies should compare the association between gender and empathy using different measures of empathy. The relationship between gender and empathy has been attributed to biological, as well as learned social and cultural factors [60,61]. The lack of female-bias in our and other analyses may indicate that the influence of these factors vary in different populations [62]. Further empirical data may be needed to determine the involvement of various facets of gender in GP physician empathy, ideally among societies with varying degrees of gender equality and gender roles among GPs.

Our results indicate that GPs who are not employed outside of clinic have a lower likelihood of having high empathy scores. Since empathy may motivate altruistic and prosocial behavior, empathic physicians may devote more time to employment that involves this type of behavior, such as teaching or volunteering outside of clinic [63,64]. In addition, empathic physicians may be more effective in balancing their clinical roles and therefore have more time and resources to participate in outside of clinic employment [39]. Further studies that analyze relationships between specific employment activities and empathy should be pursued. 

There was no relationship between the measured level of job satisfaction and empathy score in this survey, despite previously documented associations in the literature [12,40]. This could be accounted for, in part, by the fact that the clear majority of GPs in this population were at least somewhat satisfied with their jobs. Without a more uniform distribution of job satisfaction in survey participants, it is difficult to effectively evaluate this relationship.

GPs who stated a strong importance of the physician-patient relationship to job satisfaction had a greater than four-fold likelihood of being a high empathy scorer. GPs often report their most gratifying moments involve relationships with their patients, especially if the GPs demonstrate empathy [5,65]. As expected, importance of interaction with colleagues was also associated with high empathy scores, likely because interpersonal interaction and communication with others is closely tied to empathy. Importance of intellectual stimulation to job satisfaction predicts higher empathy scores in GPs as well. The JSE-HP measures physician empathy as a cognitive attribute that requires understanding patients’ perspectives, a task that may improve clinical competence through its intellectual stimulation [26]. Therefore, being highly empathetic may provide an additional layer of complexity that is rewarding to those who gain pleasure in intellectual pursuits.

## 5. Strengths and Limitations

A strength of this paper is that it explores empathy specifically in a population of GPs, who in their role as gatekeepers in the healthcare system, have a significant impact on patient care [48]. Much of the current work studying physician empathy is done in populations of medical students, who have less involvement and responsibility in the longitudinal care of patients. As empathy is related to career experience, burnout, and job satisfaction, it is difficult to extrapolate those results to apply to well-established physicians [12,33].

Another strength of this study is that the sample comprised as much as 13.5% of the population of 3436 GPs from the entirety of Denmark in 2017, while previous studies analyzing empathy in Danish physicians focused on one region [28,66]. It is also a strength that this study used stratified, rather than simple random sampling with respect to urban/rural status and practice type. Still, it is a limitation that non-respondents (61.2%) reduced the balanced proportional representativeness of the subsamples. This type of selective non-response may impede valid inference. In addition, sampling stratification was limited only to practice type and location, while other GP characteristics that were not included may have created bias.

Using the JSE-HP to measure physician empathy offers both strengths and limitations to this study. Preliminary validation of the Danish-translated version of the JSE-HP in the Danish context allows for its use in Denmark. However, it has not been as extensively evaluated as the original English version has [28]. Further work should be done in examining the psychometrics of our and other samples to further validate the JSE-HP for use in Denmark.

The JSE-HP functions as a self-report measure, which creates some limitations to its use. Individuals taking a self-report survey may be dishonest to give a good impression, but this has not been shown to occur to a large extent with the JSE-HP [27]. Additionally, the scale does not directly measure physician behaviors or patient-perception of clinician empathy. While a correlation between GP self-reported empathy and patient-perceived GP empathy has been documented, most studies show little or no association between the two [57,67,68]. The use of a neuropsychological evaluation of participants would be less susceptible to self-report bias and may also provide insight into the underlying mechanisms responsible for empathy [60,69]. Future studies that examine the link between physician self-reported empathy and more objective measures of empathy can help elucidate their association.

The JSE-HP mainly assesses the cognitive and behavioral components of empathy. This focus helps to effectively differentiate empathy from sympathy, which involves directly feeling and experiencing a patient’s suffering, and therefore may result in physician burnout and compassion fatigue if used excessively [70]. However, affective empathy seems to be related to cognitive empathy, and also important to clinical care [71]. Therefore, including alternative measures of physician empathy that involve the affective component, qualitative measures, and direct observation may have added strength to our analysis [72]. Ideally, future studies should incorporate multimodal techniques to measure physician empathy, which can add to the empathy literature.

The absence of data regarding biological correlates of health and more appropriate assessments of health among study participants is a limitation. Biologic correlates of health, such as cortisol levels, and markers of systemic inflammation have been shown to be associated with an individual’s empathy levels [73,74]. In addition, studies indicate that physicians’ health and experiences with personal illness may be related to their empathy levels as well [75,76]. Our study did not adjust for participants’ health because it was unrealistic to access this type of sensitive information through additional assessments or via registers. However, we admit that it would be ideal to do so.

Proponents argue that the advantages of using a dichotomized dependent variable and covariates are to reduce the effect of: (a) random errors in the measurement, (b) erroneous self-report responses (c) distribution characteristics (e.g., skewed). It also allows for a simpler interpretation of the link between the empathy score and covariates in terms of odds ratios. However, these advantages should be seen in relation to the limitation and disadvantages of dichotomizations (e.g., reduced variation in the data).

The field has not identified a theoretical, meaningful cut-off for the JSE-HP. Thus, the distinction between “high-scorers” and “low-scorers” was determined by the mean score (120) of a pre-existing study [20]. Results using this cut-off were consistent with a univariate, continuous analysis of empathy scores that was not included in this study. This indicates that our selected cutoff may have been appropriate. Further studies should establish specific cutoffs among different populations of physicians to allow for better determination of high and low empathy scores.

Job satisfaction was not measured using a validated psychometric tool, which served as a limitation. Finally, the present cross-sectional, multivariate analysis cannot ascribe causation between empathy and the examined characteristics. Future studies should include longitudinal analyses of physicians and their empathy levels, as well as interventions that aim to improve physician empathy among GPs.

## 6. Conclusions

Albeit in general high, we observed substantial variation in physician empathy levels among this population of Danish GPs. This variation is positively associated with values of interpersonal relationships and interaction with colleagues, and negatively associated with middle age and lack of outside employment. There is room to increase GP physician empathy via educational and organizational interventions and consequently to improve healthcare outcomes. The groundwork laid by quantifying and qualifying variation in physician empathy can help in the development of targeted interventions that may improve empathy in subsections of the GP population.

## Figures and Tables

**Table 1:** Random Strata Distribution.

Stratum	% of GPs in Population	# of GPs per Strata
Non-partnership practice and rural municipality	5.91%	70
Non-partnership practice and heterogeneous municipality	7.83%	93
Non-partnership practice and urban municipality	15.21%	182
Partnership practice and rural municipality	15.65%	187
Partnership practice and heterogeneous municipality	32.91%	394
Partnership practice and urban municipality	22.49%	269
Total	100%	1195

GP: general practitioner.

**Table 2:** Empathy scores, demographics and professional characteristics among Danish GPs.

Characteristic	Mean (SD)/Percentage	CV	p5	Median	p95
Empathy Score	117.85 (10.09)	0.09	99	118	135
Demographic Characteristics					
Physician Age	54.91 (7.86)	0.14	42	55	66
Gender					
Male	53.4%				
Female	46.6%				
Professional Characteristics					
Practice Location					
Urban practice	49.3%				
Rural practice	17.2%				
Mixed practice	33.4%				
Practice Type					
Partnership practice	72%				
Non-partnership	28%				
Employment Outside of Clinic					
Yes	54.5%				
No	45.5%				
Years since GP specialization	19.08 (8.27)	0.43	7	19	32
Years in present practice	17.19 (16.74)	0.97	4	15	31

SD: standard deviation; CV: coefficient of variation.

**Table 3:** Job satisfaction characteristics among sample of Danish GPs.

Characteristic	Mean (SD)/Percentage	CV	p5	Median	p95
Job Satisfaction					
Somewhat or very satisfied	79.7%				
Neutral	10.3%				
Somewhat or very unsatisfied	9.9%				
Contribution of Medical Practice Factors to Job Satisfaction ^±^					
Physician-patient relationship	6.17 (0.81)	0.13	5	6	7
Intellectual stimulation	5.65 (1.06)	0.19	4	6	7
Interaction with colleagues	5.41 (1.40)	0.26	3	6	7
Economic profit	4.92 (1.24)	0.25	2	5	7
Prestige	3.71 (1.5)	0.41	1	4	6

^±^ These items were rated on a 1–7 Likert scale, with 7 representing strongest contribution to job satisfaction. SD: standard deviation; CV: coefficient of variation; p5: 5th percentile; p95: 95th percentile.

**Table 4:** Logistic regression: Odds ratios for High/low empathy scores for Danish GPs.

Characteristic	Odds Ratio (95% CI)	*p*-Value	Reference Group
Demographic Characteristics			
Gender			
Male	0.91 (0.59, 1.41)	NS	Female
Age			
45–54	0.44 (0.21, 0.95)	0.036	*35–44*
55–64	0.65 (0.26, 1.64)	NS	*35–44*
65+	0.74 (0.23, 2.35)	NS	*35–44*
Professional Characteristics			
Practice location			
*Urban*	1.01 (0.65, 1.57)	NS	*Heterogeneous*
*Rural*	0.93 (0.52, 1.68)	NS	*Heterogeneous*
Practice type			
Non-partnership	1.51 (0.93, 2.46)	NS	Partnership
Employment Outside Clinic			
*No*	0.59 (0.38, 0.91)	0.016	*Yes*
Years since GP specialization			
14–22	1.19 (0.60, 2.38)	NS	*0–13*
23+	1.15 (0.47, 2.84)	NS	*0–13*
Years in present practice			
11–19	0.92 (0.49, 1.740	NS	*0–10*
20+	0.68 (0.31, 1.48)	NS	*0–10*
Job Satisfaction Characteristics			
Job Satisfaction			
Somewhat or Very Satisfied	0.95 (0.57, 1.69)	NS	*Neutral satisfaction or less*
Contribution of Medical Practice Factors to Job Satisfaction			
Physician-patient relationship			
*High (6–7)*	4.30 (2.14, 8.64)	<0.0001	*Low and Medium (1–5)*
Prestige			
*High (6–7)*	0.91 (0.48, 1.74)	NS	*Low and Medium (1–5)*
Intellectual stimulation			
High (6–7)	1.53 (0.99, 2.37)	0.053	*Low and Medium (1–5)*
Interaction with colleagues			
High (6–7)	1.90 (1.20, 3.01)	0.006	*Low and Medium (1–5)*
Economic profit			
High (6–7)	1.09 (0.72, 1.67)	NS	*Low and Medium (1–5)*
Number of respondents (N)	464		
Number of groups	406		
Wald Chi^2^	42.03	0.0002	
Variance inflation factor (mean)	2.24		
Intraclass correlation coefficient	<0.0001	0.497	

NS = not significant (*p* > 0.05). CI: confidence interval.
